# Improving Hand Hygiene Compliance of Intensive Care Unit by Using Pender's Model

**DOI:** 10.4314/ejhs.v31i3.12

**Published:** 2021-05

**Authors:** Nasrin KHosravi, Ali Alami, Mohammad Hasan Aelami, Shahla KHosrovan

**Affiliations:** 1 Department of Community Health Nursing & Management Nursing, School of Nursing, Gonabad University of Medical Sciences, Gonabad, Iran; 2 Department of Social Medicine, Social Determinants of Health Research Center, School of Public Health Faculty, Gonabad University of Medical Sciences, Gonabad, Iran. Orcid ID: https://orcid.org/0000-0002-8882-3110; 3 Department of Pediatrics, School of Medicine, Antimicrobial Resistance Research Center, Basic Sciences Research Institute, Mashhad University of Medical Sciences, Mashhad, Iran; 4 Department of Community Health Nursing & Management Nursing, School of Nursing, Social Determinants of Health Research Center, Gonabad University of Medical Sciences, Gonabad

**Keywords:** Hand Hygiene, Hospital Infections, Pender Model, Intensive care unit (ICU), self-assessment

## Abstract

**Background:**

Hand hygiene (HH) compliance is an effective behavior in controlling hospital-acquired infection because the hand is the main means of transmitting infections in patient-medical staff communication as well as the inanimate environment. This study aimed to explore the effect of applying Pender's Health Promotion Model on the HH compliance of intensive care unit staff.

**Methods:**

This quasi-experimental study with a single research group was conducted from January to July in 2019. The required data were collected from 90 staff of the intensive care units of Imam Reza Hospital in Mashhad, Iran through 1796 and 2343 opportunity of monitoring before and after the intervention. The data collection instruments were a standard HH observation form and a researcher-made HH questionnaire in the light of Pender's health promotion model. The data were statistically analyzed in SPSS using Paired-samples T-test and Chi-squared test.

**Results:**

The mean age of the 90 included participants was 35.92 (± 6.5) years and the mean length of their work experience was 10 (±1.5). The hand hygiene index rose from 23% before the intervention to 41.4% after the intervention (p=0.001). Moreover, statistically significant differences were found in moments after touching surroundings (p=0.001), before and after touching a patient (p=0.001), and also in perceived barriers (p=0.015), interpersonal influences (p=0.008) and situational influences (p<0.001).

**Conclusion:**

Pender's model showed to have improved the staff's HH compliance as a professional behavior.

## Introduction

Hand hygiene (HH) which involves a complete hand rub (HR) with alcoholic solutions or hand wash (HW) with water and soap is a simple (1), practical and effective solution for cutting down on the rate of hospital-acquired infection (2). It also plays a key role in lowering medical costs and provides more access for other patients to medical services (3). Consequently, HH is not only at the core of preventive measures against hospital infections (4) but is also a key intervention in preventing the transmission of infectious and contagious diseases in the medical system (5). Similar to a study in Vietnam, increasing the rate of HH practice for 3.2% led to a 5% decrease in hospital-acquired infections. The cost of HH compliance is less than 1% of the cost of hospital infections (6).

Over 20 thousand hospitals, until 2018, ran a campaign to show their commitment to the promotion of HH with this moto: “Clean hands save life” (4). However with all efforts put in highlighting HH in medical healthcare, the total compliance rate of HH worldwide has averaged 38.7% (7). Before touching a patient (bef-pat) and after body fluid exposure risk (aft-b.f) moments, respectively, showed to account for the lowest and highest percentages in the five moments investigated in HH (8).

A myriad of factors have been recognized as the barriers to the progress of accepting HH. These factors are related to the patient, quality of detergent materials, personnel especially nurses (9,10), physical environment (10) and inefficiency of hospital authorities and management (11,12). Since knowledge is not effective enough to change beliefs, the existence of positive behavioral beliefs and strong controller beliefs is essential. Thus, the use of such strategies as mixed interventions is recommended (13). Among the existing models, those exploring the underlying factors of HH seem to be better frameworks than controlled or planned models (14).

Pender's health promotion model (HPM) in nursing domain is a comprehensive model that has proved effective in integrating nursing and behavioral sciences approaches. In this model, there are three categories of factors known to be associated with healthy behaviors, for promotion of health. These include: prior related individual characteristics and experiences, personal factors, behavior-specific affects and perceptions (perceived barriers, benefits, self-efficacy, interpersonal influences, situational influences and behavior specific cognition and affect (positive/negative)) and behavior outcomes (commitment to plan, patterns and health promotion behavior). These together influence the health promotion beliefs directly or indirectly (15). Therefore, this model used to predict and explain health behavior and also forms the core of interventions, because they cover issues that are modified through interventions (16). Although the effect of HPM on changing behaviors related to personal health has been investigated before (17), based on our review, the effect of using this model on hand hygiene as a professional behavior has not been investigated. Accordingly, the present researchers aimed to explore the effect of a hospital-based intervention in the light of Pender's health promotion model on the intensive care unit staff concerning the HH practice as a professional behavior.

## Materials and Methods

**Study design and setting**: This present quasiexperimental study with pre- and post-test design, without control group research was conducted from January to July 2019 in the medical and general ICU of Mashhad Imam Reza Hospital in Iran.

**Study population and eligibility criteria**: The study population were physicians, nurses, nurse assistants and servant custodian who worked in the internal ICU and General ICU departments. The inclusion criteria were passed a hands-on health education course at least once in past year and willingness to participate in research and signing informed consent to participate in the research.

The exclusion criteria were dissatisfaction with continuing to participate in the research project, and skin allergy to hand sanitizer or alcohol.

**Sample size and sampling**: In this study, we used multistage sampling. In the first, due to the similarities of work and duties, the medical and general ICU was selected from 8 intensive care units through a purposive sampling method. Then, the list of attendants was prepared in two distinct sections which distinguished their ranking: physician, nurse, nurse assistant and servant custodian. The percentage of each group in the total population was also estimated and reported. Subsequently, the list of volunteers for participation in the research was prepared. If the population size was larger than the selected sample, the sampling was done as simple randomized. In the physician and custodian rankings, as the sample size was the same as the number of participating volunteers, the sampling was done as a census.

Previous studies showed a 30% rate of HH compliance among Imam Reza Hospital staff (18). Hang in Vietnam reported an increasing rate of HH compliance, from 25% to 57% (19) and the global rate of the same variable as 38.7% (20). Thus, considering the prevalence rate of HH compliance by the staff (30%) and expecting to increase it to 50% after the intervention (CI=95% and power of 80%), the required sample size was estimated as 83 and increased to 90 in order to control potential problems.

The HH practice (HW& HR) was observed for at least 5 opportunities (opp) on 3 moments. These were 1796 and 2343 cases before and after the intervention, respectively.

**Data collection instrument**: The standard WHO “HH monitoring form” - To collect the required data to determine the HH index, the standard HH monitoring form developed by WHO was employed which involved 5 situations in hospital environment including: bef-pat, before clean/aseptic procedure (befasept), aft-b.f, after touching a patient (aft-pat) and also after touching patient surroundings (aft.p.surr) (21). This form was completed through observing the behavior, and to estimate the acceptance rate of HH, the following formula was used:

$$\mathrm{HCR}=\left(\frac{\mathrm{TOTHR}+\mathrm{TOTHW}}{\mathrm{TOT}\;\mathrm{OPP}}\right)\times 100$$

**HH researcher-made questionnaire**: Moreover, to develop the intervention program, an HH researcher made questionnaire was used, which consists of 46 items, based on perceived barriers, benefits, self-efficacy, interpersonal influences, and situational influences from the Pender’ health promotion model. The items were to be rated on a 4-point Likert scale: 1 (strongly disagree), 2 (disagree), 3 (agree) and 4 (strongly agree).

In order to assess the content validity, a researcher-developed hand hygiene questionnaire was sent to specialized university professors in the field of community health with practical experience based on the Pender model and in the field of hand hygiene, of whom only seven people completed it. Then, CVR and CVI were calculated according to the data obtained from the questionnaire. In addition, CVR was determined as 0.99 using Lawshe's table, and CVI was calculated as 0.79. The questionnaire was revised based on the suggestions and proposed modifications. In order to test the reliability of the questionnaire, Cronbach's alpha was used, as indicated in Table 1.

**Table 1:** Cronbach's alpha for constructs of hand hygiene questionnaire based on Pender model

Constructs	Number of questions	Cronbach's alpha	Min Score	Max score
Perceived barriers	13	0.713	13	52
Perceived benefits	10	0.733	10	40
Perceived self- efficacy	12	0.801	12	48
Interpersonal influences	5	0.745	5	20
Situational influences	6	0.701	6	24
Total	46	0.738	46	184

**Intervention plan**: The hand hygiene practice was monitored using HH monitoring form as well as the Pender's model-based questionnaire before and after the intervention. It is noteworthy that before the intervention and 4 weeks after that, to control the observer's presence effect and monitoring precision, the data collection was repeated two times.

The interventions were implemented during May 2019 as presented below. Yet, face-to-face act of reminding continued until the end of July.

Self-assessment framework tool was considered. The Hand Hygiene Self –Assessment Implementation Framework of WHO is a systematic tool and is divided into five components (system change- training and education- evaluation and feedback- reminders in the workplace – institutional safety climate. These tools may be useful when developing an action plan and intervention to address areas identified as needing improvement (21). Therefore, the researchers used it as a guide for categorization of interventions and develop the program in five components self - assessment based on the information obtained from the researcher's questionnaire.

1. System Change: arrangements with authorities to support the project, help with the supplies, check the pretty of alcohol solution via a standard form, test the effectiveness of alcohol solution via microbial tests, change in the alcohol solution and liquid soap, provision of paper towel, replacement of sink tap, change in the place of alcohol solution, gloves supply.

2. Education and training: Within one session face-to-face group training, which took 2 hours and then for 2 weeks, the staff and authorities of the units were trained on how to practice HH and its new process correctly. They were also instructed on how to distribute pamphlets and how to provide face-to-face feedback. While training on appropriate HH practice, attention was drawn to the perceived barriers less skin damage and superiority of alcohol solution to soap, perceived benefits such as less transmission of infections to family and perceived self-efficacy such as the impossibility of avoiding untrimmed nails and jewelries in complying with HH guidelines.

3. Reminders in the workplace: With an emphasis on interpersonal influences and situational influences, reminder posters were changed; different reminders were produced; a commitment to HH poster was developed and put up, then signed by all groups; well-known personnel with respect to HH were singled out in the unit; the 2019 year motto poster was put up; face-to-face act of reminding was done by the nurse to physician or vice versa as well as the trained staff.

4. Evaluation and feedback: HH practice was monitored face-to-face or through textmessaging for all participants. As the participants were unwilling to see their names on the board (seen by all) for the result, the resultant diagram of HH practice was drawn and then displayed for each group on the board.

5. Institutional satiety: the start date of the project was sent through the automation system to the staff of public sections, and the required arrangements were made by the authorities to be present on the day the project began to sign the letter of commitment and support and follow-up the provision of alcohol solution for hand rub to be used by the staff.

The trained team referred to the hospital unit in February 2019 and monitored the HH practice of the staff for 10–30 minutes in each work shift (the peak time for HH) through direct observation on 5 moments to do so: bef-pat, befasept, aft-b.f, aft-pat and aft.p.surr (21).

**Data analysis**: The data collected through the questionnaires were entered SPSS16.0 for the required statistical analyses. For descriptive statistics, mean and frequencies were used. For inferential statistics, paired-sample *T-test* and *Chi-squared* test were sued. To test the normality of distribution, *Kolmogorov-Smirnov* test was run. The level of statistical significance was set at *P<0.05*.

**Ethical and research approvals**: This research project was approved by the committee of ethics at Gonabad University (ID code: IR.GMU.REC.1397.055). All the required arrangements were made with the participants and those involved in the research context. Their participation was upon consent, and they could leave the study upon their will.

## Results

The mean age of the participants was 35.92 (±6.5) years (min=26, max=53). The mean length of their work experience was 10 (±1.5) (min=2, max=27). The other demographic information is presented in Table 2.

**Table 2:** Demographic information of research sample

	Variable	N	%
Educational status	Under diploma	2	2.2
	Diploma	23	25.6
	Bachelor	54	60
	Masters	5	5.6
	Fellowship	6	6.7
Occupation	Nurse	59	65.6
	Doctor	6	6.7
	Assistant nurse	17	18.9
	Servant	8	8.9
Shift	Morning	13	14.4
	Evening	4	4.4
	Night	12	3.3
	Circulation	61	67.8
Sex	Male	34	37.8
	Female	56	62.2

Moreover, the extent to which HH behavior was shown by the participants is presented overall and on 5 distinct moments in Table 3. Changes in the constructs of Pender's model before and after the intervention are indicated in Table 4.

**Table 3:** Comparison of hand hygiene index before and after the intervention

Variables	Action Hand Hygiene	Before Intervention		After intervention		p

		n	%	n	%	
Total HH compliance	Yes	411	22.88	969	41.35	0.001*<
	No	1385	77.1	1374	58.64	
Bef-pat	Yes	21	3.2	107	15.78	0.001*<
	No	618	96.7	571	84.21	
Bef-asept	Yes	40	15.8	31	19.6	0.403*
	No	213	84.1	127	80.4	
Aft-b.f	Yes	38	100	68	100	1=
	No	0	0	0	0	
Aft-pat	Yes	221	42.5	461	59.17	0.001*<
	No	499	57.5	318	40.8	
Aft-pat.surr	Yes	91	26.3	306	46.3	0.001*<
	No	255	73.6	354	53.36	

* Chi-squared test

□ Fishers exact test

**Table 4:** The mean of Pender' Model structures related hand hygiene before and after intervention

Variable	Before intervention		After Intervention		T test
	
	mean	SD	mean	SD	
Perceived barriers	29.88	7.08	27.9	5.03	P= 0.015
Perceived benefits	29.86	4.21	29.12	4.30	P= 0.198
Perceived self-efficacy	38.63	5.63	38.37	4.69	P= 0.698
Interpersonal influences	13.66	2.93	14.68	2.29	P= 0.008
Situational influences	16	1.14	17.77	2.34	0.001P<

## Discussion

The present research aimed to explore the effect of a pender's hospital-based intervention on the HH practice among intensive care unit staff.

Accordingly, a comparison of the HH compliance before and after the intervention which lasted for three months would show the effectiveness of the intervention. The result showed an increase in the HH compliance from 23% to 41.4% which shows a change for 18.4%. In similar works of research such as the study in Iran (22), over a year's time, HH practice rose in prevalence from 29.3% to 70%. In another study, Anh (23) in Vietnam observed an increase in the same prevalence rate from 25.7% to 57.5% within 5 years' time. Moreover, in some other research in India, Chakravarthy (24) found an increase in the prevalence of HH practice from 36.9% to 74.8% after 7 years. The above mentioned body of research were conducted in 2010 in the light of the HH self-assessment framework (HHSA framework 2010). However, the present research not only used HHSA framework but also used the Pender's health promotion model for a comprehensive attention to the relevant causes of defective hand index (25) and attention to the behavioral causes of HH (26).

Although in the body of research reviewed, HH index showed an increase, the longer duration of them and simultaneously the probable difference in scoring and index determination is considered. Furthermore, in his research, Watson reported an increase in HH compliance from 25% to 51% in a one-year study and a focus on education. According to this research, although the effect of education and awareness-raising can be confirmed (27), ignoring the effect of other factors such as contextual barriers on behavior change can lead to the lengthening of the effect, as observed in the present research.

In the present research, HH practice bef-pat was the lowest (3.2%). The same value was 65% in the study by Helder (28) . In our research, this value rose to 15.4% within 3 months in their research, Helder et al (28), after four years and a focus on education managed to increase this index from 65% to 88%. Despite the lower percentage of change in the present research marked by less change in time can probably relate to more comprehensive changes concerned with primary investigations of a comprehensive model.

It is noteworthy that in the present research, the rate of HH practice aft- b.f risk, before and after the intervention was 100. However, in the study by Hang et al. (19) in Vietnam this value rose from 57 to 78%. In the work of research by Piers et al in Argentina (29), despite the identification of barriers to HH behavior, planning and intervention based on WHO selfassessment with an emphasis on education, the hand hygiene practice within a year rose from 66 to 75%. This difference can be explained by adherence to the removal of secretions as the cleaning of “nijasah /unclean” which is a taboo in Islam. This would point to the necessity of attention to beliefs and their effects on behavior which confirms the emphasis in Pender's health promotion model (15).

The results of the constructs of perceived barriers, the interpersonal influences and situational influences had significant changes before and after the intervention. The intervention was designed so that perceived barriers based on the model were decreased as the quality of hand alcohol and the sink valve were improved. Also, the score of interpersonal influences was improved by social support of employees to each other, improvement of norms and proposed behavioral models. Finally, the score of situational influences was improved by providing an environment demanding behavior change and positive conditions for change.

In the present research, no statistically significant difference was observed in the perceived self-efficacy and benefits scores before and after the intervention. Self-efficacy points to one's confidence in one's own capability to behave successfully and this would motivate him/her to make more attempts. Besides, more benefits perceived in performing or changing a certain behavior makes it possible to remove barriers better (15). While the body of research on Pender's health promotion model have often incorporated these two constructs in order to change personal health behavior which is more likely to perform and bring with it certain benefits (30), however, in the present research HH behavior was taken as a healthcare behavior and a professional and organizational performance. Expecting this behavior would benefit patients, and its performance would comply with certain organizational rules and regulations including financial *and* managerial conditions. Thus, it seems that to promote these two constructs, not only should patient's conditions be considered, but also the perceived personal benefits need to be taken into account, such as external motivator and provision for the persistence in an organizational professional behavior such as the control of work load.

The limited time of the study, its implementation on a limited number of intensive care unit staff, data collection procedure which involved observation and self-report, impossibility of changing personal and environmental barriers and problems, no measurement of the effect of intervention on controlling hospital-acquired infections and the role of a health-promoting hospital on social outcomes were among the limitations of the present study.

The present research approached HH as a professional behavior. The overall findings revealed that the intervention informed by Pender's health promotion model managed to increase the prevalence of HH practice as a professional behavior in general intensive care unit despite changes in perceived benefits and self-efficacy constructs. Moreover, the present research can help other similar interventional works of research, as it used a comprehensive model and increased the required knowledge for describing hand hygiene practice and its correlates. It also developed and introduced a multi-dimensional interventional program.
